# Bisphenol A Exposure Induces Small Intestine Damage Through Oxidative Stress, Inflammation, and Microbiota Alteration in Rats

**DOI:** 10.3390/toxics13050331

**Published:** 2025-04-23

**Authors:** Kai Wang, Juan Tang, Dan Shen, Yansen Li, Kentaro Nagaoka, Chunmei Li

**Affiliations:** 1 College of Animal Science and Technology, Nanjing Agriculture University, Nanjing 210095, China; wk@stu.njau.edu.cn (K.W.); tangjuan@ntu.edu.cn (J.T.); t2022067@njau.edu.cn (D.S.); liyansen@njau.edu.cn (Y.L.); 2 Department of Occupational Medicine and Environmental Toxicology, Nantong Key Laboratory of Environmental Toxicology, School of Public Health, Nantong University, Nantong 226019, China; 3 Cooperative Department of Veterinary Medicine, Tokyo University of Agriculture and Technology, Tokyo 183-8538, Japan; nagaokak@cc.tuat.ac.jp

**Keywords:** bisphenol A, intestinal barrier, oxidative stress, gut microbiota, toxicological impact

## Abstract

Bisphenol A (BPA), a widespread environmental contaminant used in plastics and resins, poses significant health risks due to its endocrine-disrupting properties and potential for inducing intestinal toxicity. This study explored the toxicological effects of BPA on the small intestine of rats, focusing on the duodenum, jejunum, and ileum. Histopathological evaluation revealed that the duodenum experienced the most severe structural damage, including villous atrophy, epithelial shedding, and mitochondrial degeneration. BPA exposure disrupted oxidative stress homeostasis by reducing superoxide dismutase activity and increasing malondialdehyde levels, along with upregulating antioxidant-related genes like *GPX2* and *HO-1* upregulated, indicating lipid peroxidation and oxidative damage. Inflammatory markers such as *IL-1* and *NFκB* were significantly upregulated, highlighting an active inflammatory response and epithelial cell apoptosis. BPA also altered lipid metabolism, with increased expression of lipogenic genes such as *SREBP-1c* and *FAS*, indicating metabolic dysregulation. Fecal microbiota analysis revealed reduced α-diversity, enrichment of pathogenic taxa like *Escherichia-Shigella*, and depletion of beneficial genera such as *Lachnospiraceae NK4A136 group*, exacerbating gut inflammation and barrier dysfunction. These findings suggest that BPA-induced small intestinal damage is driven by oxidative stress, inflammation, and gut dysbiosis, with the duodenum and jejunum being the more vulnerable segments.

## 1. Introduction

Bisphenol A (BPA) is a key chemical raw material widely used in the production of epoxy resins and polycarbonate plastics. It is commonly found in the linings of canned food containers, beverage packaging, baby bottles, water bottles, and even dental materials [1]. Under conditions such as high temperatures or acidic environments, BPA can leach from these materials into food and beverages, leading to low-dose human exposure [2]. Previous studies have demonstrated BPA’s liver and developmental toxicity in rodent models, highlighting concerns about its potential health effects [3,4].

The small intestine plays a central role in digestion and nutrient absorption while also serving critical endocrine and immune functions [5]. Goblet cells in the intestinal epithelium, originating from stem cells in the intestinal crypts, migrate upward to the villi and eventually undergo apoptosis at the villus tips. These cells secrete mucus primarily composed of mucins such as neutral mucins, sulfated mucins, and sialomucins, forming a protective layer on the epithelium [6]. The number of goblet cells increases along the small intestine from the duodenum to the ileum. Some studies have shown that maternal BPA exposure during pregnancy impairs intestinal barrier function and induces inflammatory responses in the colon of adult offspring [7,8]. Additionally, the gut is the body’s largest interface with the external environment, hosting a vast and diverse microbiota dominated by bacteria. Under normal conditions, the intestinal microbiota maintains a dynamic ecological balance. This balance is essential for regulating mucosal immune functions and supporting host health [9]. Disruptions in the gut microbiota, caused by environmental or dietary changes, can lead to dysbiosis, which has been linked to various gastrointestinal and systemic diseases.

Oxidative stress arises when reactive oxygen species (ROS) accumulate due to increased oxidative metabolism or insufficient antioxidant defenses, leading to damage to cellular macromolecules, DNA damage, and apoptosis [10]. The balance between oxidative and antioxidative systems is crucial to preventing oxidative injury. In vitro studies have reported that BPA exposure reduces superoxide dismutase (SOD) activity while increasing malondialdehyde (MDA) levels, inducing apoptosis in rat fetal liver cells [11]. In vivo, BPA is metabolized primarily in the liver through phase II enzymatic pathways, but its oxidative damage extends to tissues such as the liver, testes, and brain [12,13,14]. However, few studies have explored BPA-induced oxidative damage specifically in the small intestine.

To further elucidate BPA’s toxicological effects, this study employed a rat model to examine histopathological and histochemical changes in the intestinal mucosa across different sections of the small intestine after BPA exposure. Additionally, oxidative stress biomarkers and gut microbiota composition were assessed to provide new insights into the mechanisms underlying BPA-induced intestinal damage. This research aims to advance the understanding of BPA’s adverse effects on biological systems and support the development of mitigation strategies.

## 2. Materials and Methods

### 2.1. Reagents and Antibodies

Bisphenol A (BPA), a white powder (C15H16O2, ≥99%, CAS No. 80-05-7), was purchased from Sigma-Aldrich (St. Louis, MO, USA). Assay kits for total superoxide dismutase (SOD), malondialdehyde (MDA), catalase (CAT), and glutathione peroxidase (GSH-PX) were obtained from Nanjing Jiancheng Bioengineering Institute (Nanjing, China). Trizol reagent was supplied by Invitrogen (Carlsbad, CA, USA), while the Takara PrimeScript^®^ RT Reagent Kit and RT-PCR reverse transcription kits were purchased from Takara Bio (Dalian, China). DNA concentration was measured using the NanoDrop 1000 spectrophotometer (NanoDrop Technologies, Wilmington, DE, USA). All other reagents used were of analytical grade and produced in China.

### 2.2. Animals and Sampling

Twenty-four 8-week-old male Sprague–Dawley (SD) rats were obtained from the Qinglongshan Laboratory Animal Center in Nanjing, Jiangsu Province, China. Only male Sprague–Dawley rats were used in this study to reduce the potential confounding effects of sex hormones, which have been shown to influence BPA metabolism, oxidative stress response, and gut microbiota composition [7]. Our primary aim was to ensure consistent physiological responses and minimize hormonal variations that could affect the inflammatory and oxidative stress markers under investigation. The panel responsible for animal research at the Nanjing Agricultural University’s Institute, in conjunction with the National Health Institute’s Animal Care protocols, conducted a thorough review and subsequently approved the research methods (SYXK(Su)2021-0086). The rats were housed in a controlled environment at a temperature of 23 ± 2 °C, relative humidity of 50% ± 5%, and a 12 h light/12 h dark cycle with proper ventilation. Animals had free access to standard laboratory chow and water and were individually housed in separate cages to prevent cross-contamination of microbiota. Before the formal experiment, rats were acclimatized for one week. The 24 male SD rats were randomly assigned to four groups (*n* = 6 per group). BPA was dissolved in corn oil before administration. Rats in the treatment groups received BPA by oral gavage at doses of 0.5, 5, and 50 mg/kg body weight daily at 9:00 a.m., while the control group received an equivalent volume of corn oil. The treatment lasted for 30 consecutive days. The selected BPA doses (0.5, 5, and 50 mg/kg) were based on previous studies assessing the toxicological effects of BPA in rodents [15]. The 5 mg/kg dose approximates the human tolerable daily intake level set by regulatory agencies, whereas the 50 mg/kg dose represents a high-exposure scenario relevant to occupational or environmental contamination settings [16]. Twenty-four hours after the last gavage, the rats were euthanized using carbon dioxide inhalation followed by cervical dislocation to ensure humane and effective euthanasia. Urine and fecal samples were collected, transferred into sterile centrifuge tubes, and stored at −20 °C for ion concentration analysis and fecal microbial DNA extraction. Blood samples were collected from the abdominal aorta, allowed to clot at room temperature for 30 min, and centrifuged at 1300× *g* for 15 min at 4 °C to separate the serum, which was aliquoted and stored at −20 °C for further analysis.

Tissues from the duodenum, jejunum, ileum, and colon were harvested. Portions of these tissues were fixed in 4% paraformaldehyde and 2.5% glutaraldehyde solutions for histological and ultrastructural examination. The remaining tissues were stored at −80 °C for ion concentration measurement and RT-PCR analysis.

### 2.3. Morphological Analysis

The preserved intestinal segments were removed from the fixative and progressively dehydrated using ethanol and chloroform of ascending concentrations. Afterward, the tissues were embedded in paraffin and sectioned into thin slices with a microtome. The sections were stained with hematoxylin and eosin for histological examination. For morphometric evaluation, two transverse sections from each intestinal region (duodenum, jejunum, and ileum) were mounted on a single slide. Ten well-aligned crypt-villus units were randomly selected per sample for measurement. Villi height (VH) was determined from the tip of the villi to the base between adjacent villi, while crypt depth (CD) was measured from the trough between neighboring villi to the basal membrane. Morphometric analyses were performed using an Olympus BX51 microscope equipped with a DP70 digital camera (Olympus, Tokyo, Japan) and JD801 morphologic image analysis software.

### 2.4. Oxidative Stress Determination

SOD, MDA content, CAT, and GSH-Px enzyme activities in the duodenum, jejunum, and ileum were determined using Spectrophotometric Kits (Jiancheng Bioengineering Institute, Nanjing, China) by the manufacturer’s instructions. SOD activity was measured based on its ability to inhibit the reduction of nitroblue tetrazolium, and results were expressed as U/mL protein. CAT and GSH-PX activity was quantified by monitoring the oxidation of reduced glutathione, with results expressed in U/mg prot protein. MDA content was determined via the thiobarbituric acid reactive substances (TBARS) method and expressed in nmol/mL protein. A standard curve was used for the quantification of each enzymatic assay to ensure accuracy, and all samples were analyzed in duplicate to minimize variability.

### 2.5. Ultrastructure Observation

The jejunum samples were fixed in freshly prepared 2.5% glutaraldehyde at 4 °C for 24 h, followed by a 12 h rinse with phosphate-buffered saline (PBS). Afterward, the tissues were post-fixed in 1% osmium tetroxide (OsO_4_) for 20 min, rinsed with PBS, and dehydrated through a graded ethanol series (20% to 100%). They were then infiltrated with a 1:1 mixture of Epon resin and ethanol for 2 h, followed by 100% Epon embedding for 3 h. The samples were incubated overnight in an oven. Ultra-thin sections (approximately 70 nm thick) were cut and mounted on copper grids. Sections were stained with 2% uranyl acetate, followed by 1% lead citrate for 30 min. The ultrastructure of the jejunum samples was examined using a JEM-100CX transmission electron microscope (TEM, JEOL Ltd. Tokyo, Japan) operated at 80 kV.

### 2.6. Quantitative Real-Time PCR Analysis

Frozen RNA samples (100 mg) were rapidly transferred to a pre-cooled mortar containing liquid nitrogen and ground into a fine powder. The powdered tissue was quickly transferred into 1 mL of Trizol reagent in a microcentrifuge tube, mixed thoroughly by inversion, and left to stand for 10 min. Following this, 200 µL of chloroform was added, mixed well, and incubated for 5 min. The mixture was centrifuged at 1600× *g* for 15 min at 4 °C to separate the phases. The upper colorless aqueous phase (approximately 600 µL) was carefully collected. To precipitate RNA, 600 µL of isopropanol was added to the aqueous phase, mixed thoroughly, and incubated at −20 °C for 10 min. The mixture was centrifuged under the same conditions for 15 min, and the supernatant was discarded. The RNA pellet was washed with 75% ethanol and centrifuged again, and the residual ethanol was carefully removed. The pellet was air-dried for 30 min in a sterile environment until it appeared transparent. Finally, 20 µL of RNase-free water was added to dissolve the RNA. For reverse transcription, the extracted RNA was converted into cDNA using reverse transcriptase (such as M-MLV or SuperScript). The reaction mixture contained specific primers, dNTPs, and appropriate buffer solutions. The reverse transcription reaction was performed according to the enzyme manufacturer’s protocol, typically at 42 °C for 60 min. The resulting cDNA was analyzed using quantitative PCR (qPCR) to measure gene expression levels. Specific primers were used to amplify target genes, and relative expression levels were calculated by comparing CT values under different treatment conditions. This allowed the assessment of gene expression differences between experimental groups. The primer sequence is shown in Table 1.

### 2.7. Fecal Microbial Community Analysis

The collected fecal samples were thawed and placed on ice, with 0.1 g of each sample weighed for DNA extraction using the PowerSoil DNA Isolation Kit (MO BIO Laboratories, Carlsbad, CA, USA) according to the manufacturer’s instructions. DNA was then purified using the Mobio PowerClean DNA Clean-Up Kit (MO BIO Laboratories, Carlsbad, CA, USA), and the purified genomic DNA was assessed via 1% agarose gel electrophoresis. For PCR amplification of the V1–V3 region, primers containing unique barcodes at the 5′ end were used to bind to the 16S rRNA V1–V3 region, with each sample assigned a distinct barcode. The primer sequences were as follows: 27F AGAGTTTGATCCTGGCTCAG and 533R TTACCGCGGCTGCTGGCA. PCR was performed using TransGen AP221-O2: TransStart Fastpfu DNA Polymerase, in a 20 μL reaction system consisting of 10 ng DNA template, 0.4 μL each of the forward and reverse primers (5 μM), 2 μL dNTPs (2.5 mM), 0.4 μL FastPfu Polymerase, and 4 μL of 5× FastPfu Buffer. The PCR amplification was conducted on an ABI GeneAmp^®^ 9700 thermal cycler (Applied Biosystems, Waltham, MA, USA) with the following program: 95 °C for 2 min (initial denaturation), followed by 25 cycles of 95 °C for 30 s, 55 °C for 30 s, and 72 °C for 30 s, with a final extension at 72 °C for 5 min. PCR products were recovered from 2% agarose gel electrophoresis, and DNA was extracted using the AxyPrep DNA Gel Recovery Kit (AXYGEN, Union City, CA, USA) with elution in Tris-HCl. The PCR products were then quantified using the QuantiFluor™-ST Blue Fluorescence Quantification System (Promega, Madison, WI, USA). Based on the initial quantification results, the samples were mixed in appropriate proportions according to the sequencing requirements. High-throughput sequencing was performed using the Roche 454 platform, with support from Shanghai Meiji Bio-Medical Technology Co., Ltd. (Shanghai, China).

### 2.8. Statistical Analysis

All data are presented as the mean ± standard error of the mean (SEM). Initially, the data were organized using Excel. For normally distributed data, a one-way analysis of variance (ANOVA) was performed using SPSS 20.0. For data that did not follow a normal distribution, a 10 g transformation was applied before performing one-way ANOVA, or non-parametric analysis was conducted directly using the Kruskal–Wallis test. Additionally, statistical analysis was performed using GraphPad Prism Version 7.0 (GraphPad Software, San Diego, CA, USA). *p* < 0.05 and *p* < 0.01 were considered indicative of significant and highly significant differences, respectively, compared to the control group.

## 3. Results

### 3.1. Bisphenol A on the Structural Integrity of Rat Small Intestine

**Figure 1:**
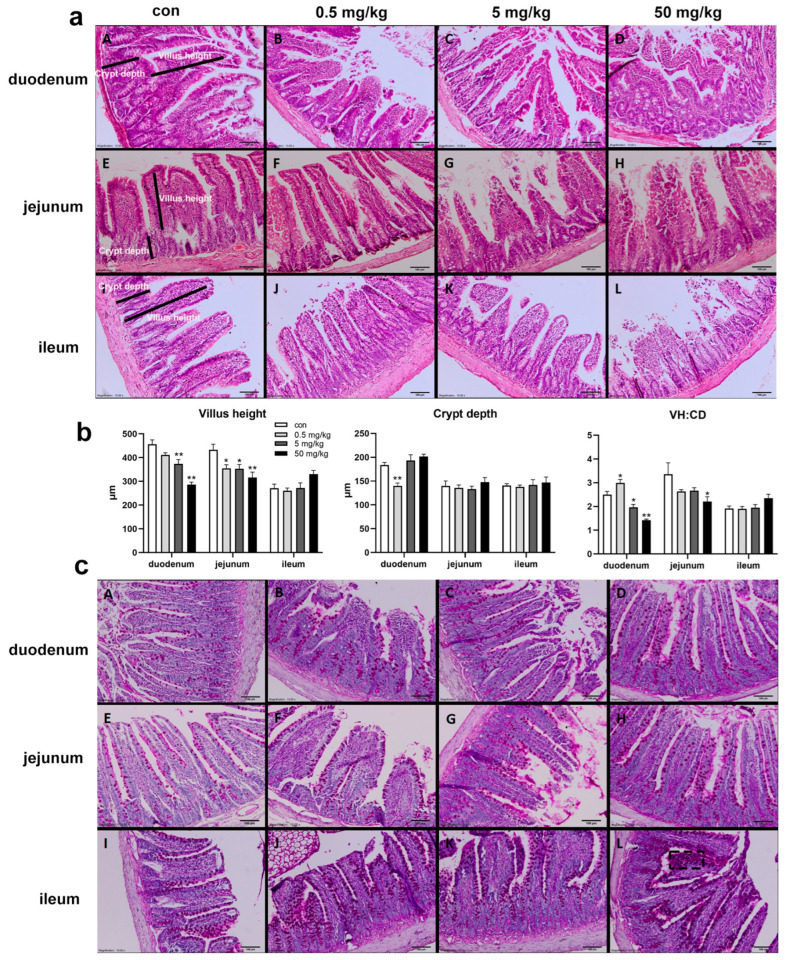
BPA exposure on the small intestine histopathological by HE and PAS staining in rats. H&E staining of duodenum X100, 100 μm: (**aA**) control; (**aB**) 0.5 mg/kg BPA-treated rat; (**aC**) 5 mg/kg BPA-treated rat; (**aD**) 50 mg/kg BPA-treated rat. The arrangement of jejunum (**a**: **E**–**H**) and ileum (**a**: **I**–**L**) annotations are consistent with that of duodenum. (**b**) Villus height, crypt depth, and ratio (VH:CD). (**c**) PAS staining; arrangement annotations are consistent with (**a**). (**cL**) The black dashed box indicates goblet cells. *n* = 6. * represents significant differences, *p* < 0.05. ** represents highly significant differences, *p* < 0.01.

The HE staining results of the small intestinal mucosa showed normal mucosal morphology with intact villi in the control group (Figure 1a). In the BPA-treated groups, varying degrees of small intestinal damage were observed, including villous injury, central necrosis, epithelial shedding, lamina propria disintegration, hemorrhage, and ulcer formation. The interstitial tissue exhibited congestion, edema, and neutrophil infiltration, with epithelial cell degeneration and necrosis at the villous tips (Figure 1a). Compared to the control group, the duodenal villus height was significantly reduced in the 5 mg/kg and 50 mg/kg BPA-treated groups (*p* < 0.05), while crypt depth was significantly increased in the 0.5 mg/kg BPA-treated group (*p* < 0.05), with no significant differences observed in other dosage groups (Figure 1b). The villus-height-to-crypt-depth ratio decreased significantly across 5 and 50 mg/kg BPA-treated groups (*p* < 0.05). In the jejunum, villus height was significantly reduced in all BPA-treated groups compared to the control (*p* < 0.05), with no significant changes in crypt depth. However, the villus-height-to-crypt-depth ratio was significantly reduced in the 50 mg/kg BPA-treated group (*p* < 0.05). BPA exposure did not cause significant changes in villus height, crypt depth, or the villus height-to-crypt depth ratio in the ileum at any tested dosage (Figure 1b). Furthermore, PAS staining under electron microscopy revealed a marked increase in goblet cell numbers in the intestinal epithelium of BPA-treated rats compared to the control group (Figure 1c).

### 3.2. Bisphenol A on the Ultrastructure of the Rat Jejunum

**Figure 2:**
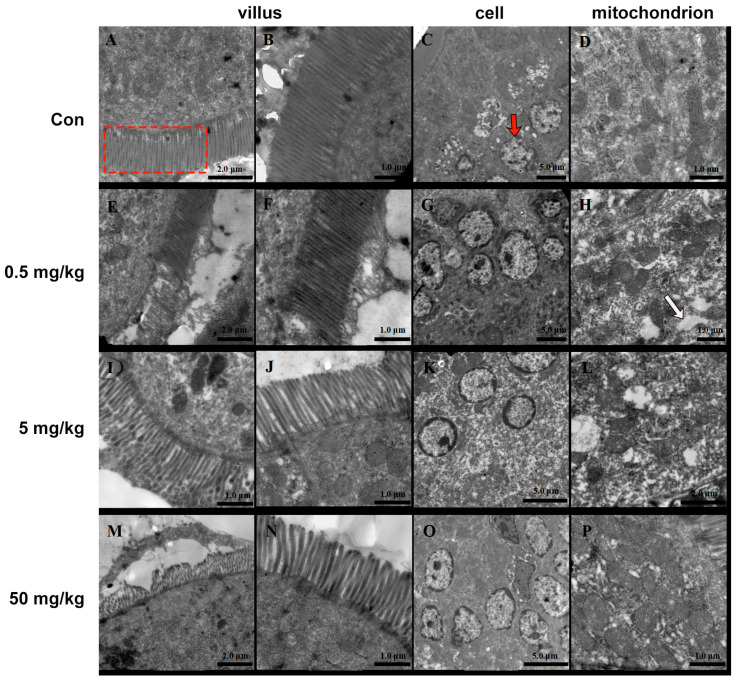
BPA on the ultrastructure of rat jejunum. (**A**–**D**) Control; (**E**–**H**) 0.5 mg/kg; BPA-treated rat; (**I**–**L**) 5 mg/kg BPA-treated rat; (**M**–**P**) 50 mg kg BPA-treated rat. The red dashed box indicates villus in A. The red arrow indicates cell in C. The white arrow indicates cytoplasmic vacuoles. *n* = 6.

The small intestinal villi of rats in the normal group were neatly arranged, with smooth surfaces and no signs of shedding or damage (Figure 2A,B). The intestinal epithelial nuclei appeared round (Figure 2C), surrounded by numerous intact mitochondria (Figure 2D). In contrast, the BPA-treated groups exhibited significant morphological alterations. The intestinal villi were markedly shortened and reduced in number and showed widened inter-villous spaces. Secretions and detached epithelial tissues adhered in sheet-like structures (Figure 2E,I,M). The 50 mg/kg BPA cellular nuclei exhibited pyknosis, reduced size, chromatin condensation, marginalization, and perinuclear cisternae expansion (Figure 2O). The number and size of mitochondria were notably reduced (Figure 2K). Additionally, cytoplasmic vacuoles and osmophilic granules were observed (Figure 2H,L,P).

### 3.3. Bisphenol A on Oxidative Stress in Rat Small Intestinal Tissue

**Figure 3:**
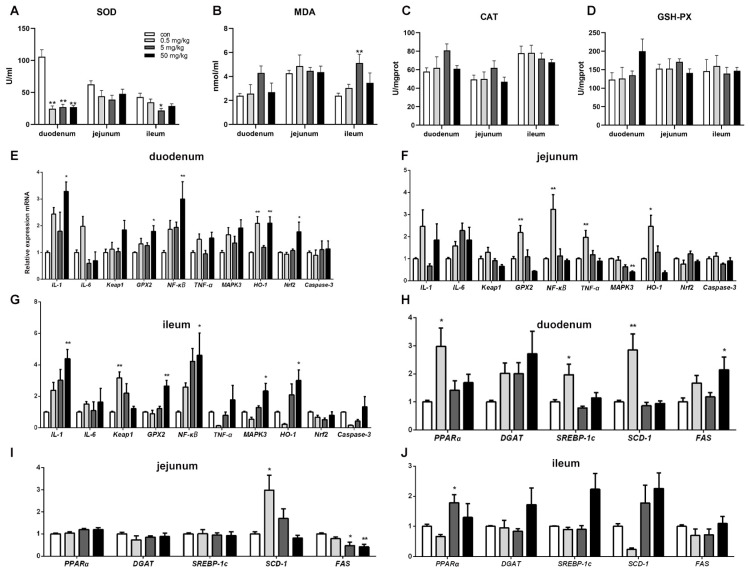
BPA on oxidative stress parameters, inflammatory and lipid metabolism genes in rat small intestine tissue. (**A**–**D**) Oxidative stress parameters SOD, MDA, CAT, and GSH-PX; (**E**–**G**) duodenum, jejunum, and ileum inflammatory genes; (**H**–**J**) duodenum, jejunum, and ileum lipid metabolism genes. *n* = 6. * represents significant differences, *p* < 0.05. ** represents highly significant differences, *p* < 0.01.

Compared to the control group, SOD activity in the duodenal tissue was significantly reduced (*p* < 0.05) across all BPA-treated groups, while other oxidative stress indicators showed no significant changes (Figure 3A). In the ileum, SOD activity was significantly decreased (*p* < 0.05), and MDA content was significantly increased (*p* < 0.05) in the 5 mg/kg BPA-treated group. However, no significant differences were observed in oxidative stress indicators in the jejunal tissue among the various treatment groups (Figure 3B–D).

### 3.4. Bisphenol A on the Expression of Oxidative Stress, Inflammation, and Apoptosis-Related Genes in Rat Small Intestinal Tissue

Compared to the control group, the mRNA levels of inflammation-regulating genes *IL-1*, *GPX2*, and *NFκB* in duodenal tissue were significantly increased in the 50 mg/kg BPA-treated group (*p* < 0.05). Additionally, HO-1 mRNA expression was significantly elevated in the 0.5 and 50 mg/kg BPA-treated groups (*p* < 0.05). The expression level of the oxidative stress-related gene Nrf2 was also significantly upregulated in the duodenal tissue of the 50 mg/kg BPA-treated group (Figure 3E, *p* < 0.05). In jejunal tissue, *GPX2*, *NFκB*, *TNF-α*, and *HO-1* expression levels were significantly increased in the 0.5 mg/kg BPA-treated group (*p* < 0.05), whereas *MAPK3* expression was significantly decreased in the 50 mg/kg BPA-treated group (Figure 3F, *p* < 0.05). In ileal tissue, the expression levels of *IL-1*, *GPX2*, *NFκB*, *MAPK3*, and *HO-1* were significantly upregulated in the 50 mg/kg BPA-treated group (*p* < 0.05), while *Keap1* expression was significantly elevated in the 0.5 mg/kg BPA-treated group (Figure 3G, *p* < 0.05).

### 3.5. Bisphenol A on the Expression of Lipid-Related Genes in Rat Small Intestinal Tissue

BPA exposure significantly influenced the expression of lipid metabolism-related genes in rat small intestinal tissue. In duodenal tissue, the mRNA levels of *PPAR-α*, *SREBP-1c*, and *SCD-1* were significantly upregulated in the 0.5 mg/kg BPA-treated group (*p* < 0.05), while FAS mRNA expression was markedly increased in the 50 mg/kg BPA-treated group (*p* < 0.05) (Figure 3H). In jejunal tissue, *SCD-1* mRNA expression was significantly elevated in the 0.5 mg/kg BPA-treated group (*p* < 0.05), whereas *FAS* mRNA expression was significantly reduced in the 5 mg/kg and 50 mg/kg BPA-treated groups (*p* < 0.05) (Figure 3I). In ileal tissue, only *PPARα* mRNA expression showed a significant increase in the 50 mg/kg BPA-treated group (*p* < 0.05), with no significant changes observed in other genes (Figure 3J).

### 3.6. Bisphenol A on Fecal Microbiota Composition in Rats

**Figure 4:**
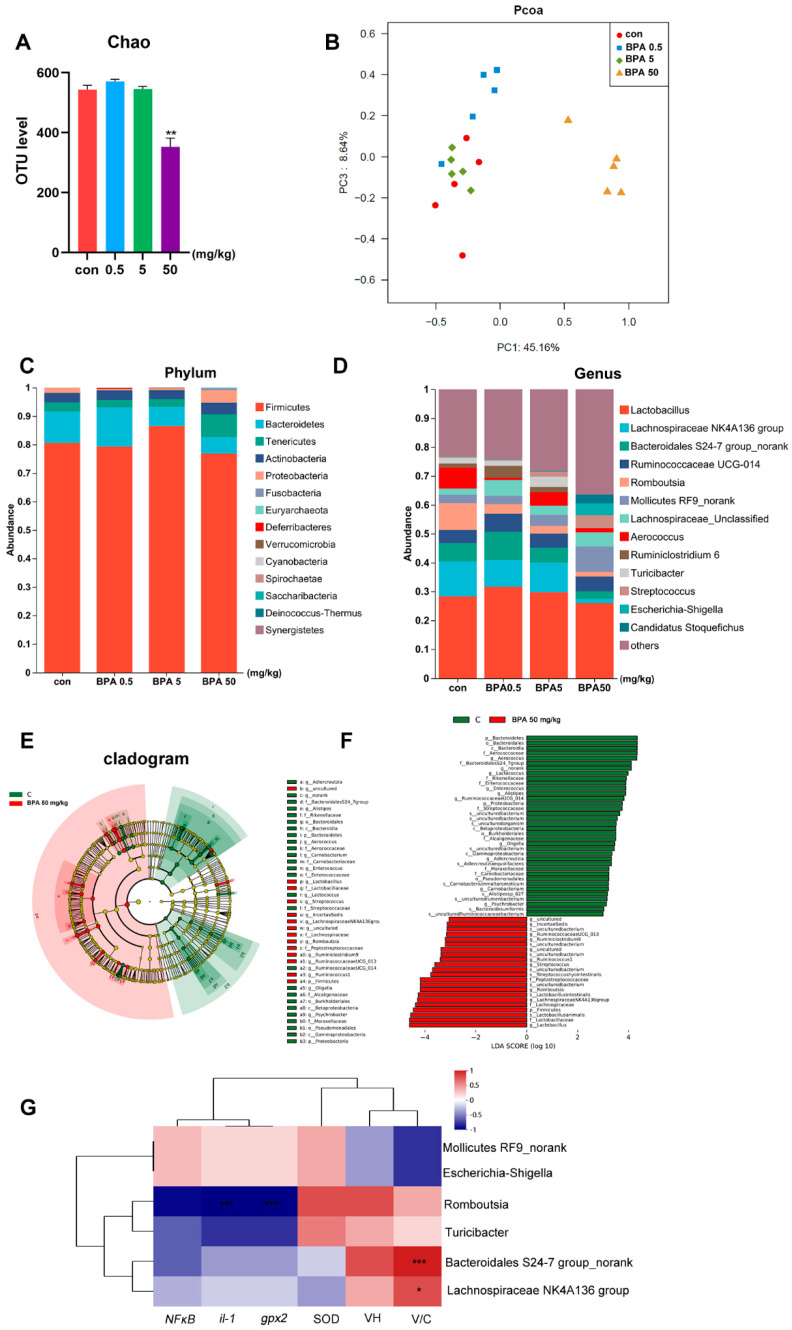
BPA on oxidative stress parameters and inflammatory and lipid metabolism genes in rat small intestine tissue: (**A**) α-diversity; (**B**) β-diversity; (**C**) phylum community; (**D**) genus community; (**E**,**F**) Lefse, LDA; (**G**) correlation analysis of differential bacteria and inflammatory factors, villous crypt ratio (V/C), and oxidative stress indicators. *n* = 6. * represents significant differences, *p* < 0.05. ** represents highly significant differences, *p* < 0.01. *** represents highly significant differences, *p* < 0.001.

Compared with the control group, the fecal microbiota of rats treated with 50 mg/kg BPA exhibited significantly reduced α-diversity and distinct β-diversity clustering (Figure 4A,B, *p* < 0.05). At the phylum level, the relative abundance of *Firmicutes* increased in the 5 mg/kg BPA group, while *Tenericutes* and *Proteobacteria* were more abundant in the 50 mg/kg BPA group. Conversely, *Bacteroidetes* showed decreased relative abundance in both the 5 mg/kg and 50 mg/kg BPA treatment groups (Figure 4C). At the genus level, BPA significantly decreased the relative abundance of *Lachnospiraceae NK4A136 group*, *Bacteroidales S24-7 group_norank*, *Romboutsia*, *Aerococcus*, *Turicibacter*, and several unclassified genera (*p* < 0.05). Meanwhile, the relative abundances of *Streptococcus*, *Escherichia-Shigella*, and *No-rank Mollicutes* increased notably (Figure 4D). Linear discriminant analysis (LDA) identified *Lachnospiraceae NK4A136 group*, *Bacteroidales S24-7 group_norank*, *Romboutsia*, *Turicibacter*, *Escherichia-Shigella*, and *No-rank Mollicutes* as the most contributive taxa (Figure 4E,F). Among these, *Lachnospiraceae NK4A136 group* and *Bacteroidales S24-7 group_norank* were significantly positively correlated with V/C, while *Romboutsia* was negatively correlated with *IL-1* and *GPX2* (Figure 4G).

## 4. Discussion

BPA exposure induces significant histopathological alterations in the rat small intestine, particularly in the duodenum. Villus height and crypt depth are critical indicators of intestinal digestive and absorptive capacity, with a higher villus-height-to-crypt-depth ratio indicating enhanced nutrient absorption efficiency [17]. In this study, BPA exposure led to reduced villus height, increased crypt depth, and a decreased villus-height-to-crypt-depth ratio in the duodenum, signifying compromised intestinal absorption. Similar structural damage, including villous shortening, increased inter-villous spaces, and epithelial cell shedding, aligns with previous findings linking BPA exposure to gastrointestinal tissue injury [18]. The observed disruption of villus architecture likely reflects impaired nutrient absorption and weakened intestinal barrier function, predisposing the gut to increased permeability and heightened susceptibility to gastrointestinal disorders, which is consistent with earlier reports. Additionally, the significant increase in goblet cell numbers following BPA exposure suggests a compensatory response aimed at enhancing mucosal defense. Goblet cells play a crucial role in maintaining intestinal homeostasis by secreting mucus that forms a protective barrier and modulates immune responses through specific and nonspecific mechanisms [19]. However, the functional consequences of this response require further investigation, particularly regarding the quality and composition of the mucus produced. Moreover, the ultrastructural examination revealed mitochondrial damage, chromatin condensation, and cytoplasmic vacuolation in epithelial cells, suggesting severe oxidative stress-induced apoptosis. These morphological changes correspond to increased expression of inflammation and apoptosis-related genes such as *IL-1*, *NFκB*, *GPX2*, and *HO-1*, indicating an active inflammatory response and cellular stress. These findings highlight that BPA exposure disrupts the structural integrity of the duodenal mucosa, impairs nutrient absorption, and compromises the intestinal barrier.

BPA exposure exerts significant damage on the structure and function of the small intestine, potentially through mechanisms involving oxidative stress and inflammation. BPA induces oxidative stress in various tissues, highlighting its toxicological effects [20]. Given the small intestine’s critical role in nutrient absorption, its epithelial cells are highly susceptible to oxidative damage [21]. BPA-induced accumulation of reactive oxygen species (ROS) can overwhelm the antioxidant defense system, primarily regulated by enzymes such as SOD and glutathione peroxidase GSH-Px. In this study, decreased SOD activity coupled with elevated malondialdehyde MDA levels indicated impaired antioxidant defense, resulting in lipid peroxidation and cellular damage. Concurrently, the expression of pro-inflammatory genes, including *IL-1* and the key inflammatory regulator *NFκB*, was significantly upregulated, indicating a robust inflammatory response triggered by BPA exposure. *NFκB* activation not only enhances the expression of inflammatory mediators but also contributes to epithelial cell apoptosis and compromised intestinal barrier integrity [22]. Moreover, the increased expression of *HO-1*, a hallmark of cellular stress response, may represent a protective adaptive mechanism; however, its prolonged activation could exacerbate inflammatory conditions. Therefore, BPA disrupts intestinal structural integrity and homeostasis by inducing oxidative stress and activating inflammatory pathways, contributing to small intestinal tissue damage.

The disruption of antioxidant defense and activation of inflammatory pathways in the small intestine following BPA exposure may further contribute to metabolic dysregulation, particularly in lipid metabolism. Lipid-related gene expression analysis revealed significant changes, especially in the duodenum and jejunum. The upregulation of *PPAR-α*, *SREBP-1c*, and *SCD-1* suggests that BPA may interfere with lipid homeostasis by promoting lipogenesis and altering fatty acid metabolism. This metabolic shift could exacerbate oxidative stress and inflammation through increased production of bioactive lipids and free fatty acids. Previous studies have demonstrated BPA’s role in disrupting metabolic pathways and promoting lipid accumulation in various tissues [23,24]. The enhanced expression of *PPAR-α*, a regulator of fatty acid oxidation, alongside *SREBP-1c* and *SCD-1*, key players in lipid synthesis, indicates a paradoxical metabolic response. This dysregulated lipid metabolism could worsen intestinal epithelial damage by amplifying inflammatory and oxidative processes. Such metabolic disturbances reflect BPA’s broader toxicological effects, contributing to a heightened risk of metabolic disorders and intestinal dysfunction. BPA-induced upregulation of genes like *PPAR-α*, *SREBP-1c*, and *SCD-1*, which regulate lipid synthesis and metabolism, could create a lipid-rich intestinal milieu that supports the growth of opportunistic microbial species while suppressing beneficial microbes [25]. Additionally, previous research has highlighted the gut microbiota’s sensitivity to environmental toxicants, including BPA, which can disrupt microbial diversity and promote dysbiosis [26,27]. Given the critical role of gut microbiota in modulating inflammation and maintaining intestinal barrier integrity, BPA’s impact on microbial composition might further intensify oxidative stress and inflammatory responses.

BPA exposure significantly disrupted gut microbial homeostasis, affecting key bacterial taxa linked to intestinal health. These alterations likely mediated the BPA-induced oxidative stress, inflammation, and lipid metabolism dysregulation observed in this study. The phylum-level changes indicated dose-dependent microbial shifts. Increased Firmicutes in the 5 mg/kg BPA group suggests potential alterations in energy harvesting and SCFA production, which could disrupt gut homeostasis [28]. Conversely, elevated Tenericutes and Proteobacteria in the 50 mg/kg BPA group point to inflammatory responses, as Proteobacteria is often associated with gut barrier dysfunction and systemic inflammation due to its endotoxin-producing potential [29]. Meanwhile, the reduced Bacteroidetes, typically linked to anti-inflammatory effects, reflects compromised gut health and metabolic imbalance. At the genus level, BPA exposure led to marked reductions in beneficial bacteria. *Lachnospiraceae NK4A136 group* plays a crucial role in producing anti-inflammatory SCFAs such as butyrate, essential for gut epithelial health [30]. Its decline likely exacerbated intestinal inflammation and contributed to reduced villus height, as supported by its positive correlation with the villus-to-crypt (V/C) ratio. Similarly, *Bacteroidales S24-7 group_norank*, another SCFA producer associated with immune modulation, decreased, possibly weakening the gut barrier and facilitating inflammation [31,32]. The reduced abundance of *Romboutsia*, a genus involved in carbohydrate metabolism and gut epithelial protection, was noteworthy [33]. Its negative correlation with *IL-1* and *GPX2* indicates that its loss may have enhanced oxidative stress and inflammatory responses, compromising mucosal defense. *Turicibacter*, commonly linked to intestinal integrity and immune regulation, also decreased, further supporting disrupted gut barrier function [34,35]. On the other hand, *Escherichia-Shigella* and *Streptococcus* were enriched, both of which are potential opportunistic pathogens. The overgrowth of *Escherichia-Shigella*, known for triggering gut inflammation through toxin production, likely intensified mucosal damage and inflammatory cascades [36]. Similarly, *Streptococcus*, although typically commensal, can induce inflammation when overrepresented, worsening gut injury [37]. BPA-induced dysbiosis, as observed in this study, could further contribute to oxidative stress-mediated damage. The reduction in beneficial taxa such as *Lachnospiraceae NK4A136 group*, and *Bacteroidales S24-7 group_norank* following BPA exposure may create a more pro-oxidant and inflammatory environment. Concurrently, the enrichment of *Escherichia-Shigella* and *Streptococcus* may exacerbate oxidative stress through the production of ROS and endotoxins [38]. This suggests that the microbial imbalance induced by BPA contributes to a feed-forward loop, where oxidative stress disrupts gut microbiota, and in turn, dysbiosis aggravates oxidative stress and inflammation, amplifying intestinal damage. In addition, BPA exposure has been associated with gut barrier dysfunction, often mediated through the disruption of tight junction proteins such as occludin, zonula occludens-1, and claudin-1. Studies have demonstrated that BPA can downregulate the expression of these key proteins, leading to increased intestinal permeability and compromised epithelial integrity [39]. The observed dysbiosis, particularly the enrichment of *Escherichia-Shigella* and *Streptococcus*, may further exacerbate barrier dysfunction by promoting inflammation and oxidative stress. The resulting impairment in tight junction integrity facilitates bacterial translocation, thereby amplifying immune responses and contributing to systemic inflammation. These findings suggest that BPA-induced alterations in gut microbiota and oxidative stress may work synergistically to weaken gut barrier function, increasing the risk of gastrointestinal disorders. Overall, BPA-induced gut dysbiosis likely plays a central role in promoting oxidative stress, inflammation, and impaired lipid metabolism through disruptions in SCFA-producing and inflammation-modulating bacteria. This microbial imbalance may serve as a mechanistic link between BPA exposure and intestinal pathophysiology. Future studies should further explore these microbial dynamics and their functional implications for gut health.

We acknowledge several limitations in this study. First, we did not perform functional analyses such as metagenomics or metabolomics, which could have provided deeper insights into the mechanistic roles of the observed microbial changes. Additionally, while we identified clear correlations between BPA-induced gut microbial shifts and intestinal pathophysiology, these associations remain observational, and future studies using interventions like microbial transplantation are needed to confirm causality. Moreover, our study focused on controlled BPA doses, but the effects of chronic low-dose exposure, which may better reflect real-world conditions, require further investigation. Despite these limitations, we believe this work makes an important contribution by being the first to comprehensively examine the effects of BPA on microbial communities across the entire small intestine. By linking microbial dysbiosis to oxidative stress, inflammation, and disrupted lipid metabolism, we provide novel insights into how BPA exposure impacts gut health.

## 5. Conclusions

In conclusion, the present study demonstrates that BPA exposure induces substantial structural, molecular, and microbial alterations in the rat small intestine, with the duodenum being the most affected segment. Histopathological and ultrastructural examinations revealed severe damage to the intestinal epithelium, characterized by villous atrophy, epithelial shedding, and mitochondrial degeneration, while molecular analysis confirmed oxidative stress, inflammation, and apoptotic responses. BPA exposure also resulted in significant dysregulation of lipid metabolism, marked by the upregulation of key lipid-related genes, which may exacerbate oxidative stress and inflammation. Moreover, BPA altered the fecal microbiota composition, promoting gut dysbiosis characterized by an increase in pathogenic taxa and a decrease in beneficial bacteria, further contributing to the observed intestinal damage. These findings suggest that BPA disrupts intestinal homeostasis through a multifaceted mechanism involving structural injury, oxidative and inflammatory responses, altered lipid metabolism, and microbial imbalance. The study underscores the importance of considering BPA’s impact on gut health and its potential role in the pathogenesis of gastrointestinal disorders and metabolic dysfunctions. Further research is needed to elucidate the precise microbial interactions and to explore potential therapeutic strategies to mitigate BPA-induced gut damage.

## Figures and Tables

**Table 1:** Primers used for quantitative real-time PCR.

Gene	Sequence (5′ to 3′)	Size (bp)
*β-actin*	F: CTAAGGCCAACCGTGAAAAGAT R: CACAGCCTGGATGGCTACGT	83
*IL-1β*	F: GCCAACAAGTGGTATTCTCCA R: TGCCGTCTTTCATCACACAG	120
*IL-6*	F: AGTTGCCTTCTTGGGACTGA R: ACTGGTCTGTTGTGGGTGGT	102
*MAPK3*	F: CTACACGCAGCTGCAGTACATC R: GTGCGCTGACAGTAGGTTTGA	153
*NF-kB*	F: CGACGTATTGCTGTGCCTTC R: TTGAGATCTGCCCAGGTGGTA	198
*TNF-α*	F: TTCCGTCCCTCTCATACACTG R: AGACACCGCCTGGAGTTCT	149
*Keap1*	F: CATCGGCATCGCCAACTTC R: GCTGGCAGTGTGACAGGTTGA	278
*GPx2*	F: CCGTGCTGATTGAGAATGTG R: AGGGAAGCCGAGAACCACTA	113
*Caspase-3*	F: AAGCCGAAACTCTTCATC R: TGAGCATTGACACAATACAC	349
*PPARα*	F: CTCGTGCAGGTCATCAAGAA R: CAGCCCTCTTCATCTCCAAG	158
*Nrf2*	F: CAGTGCTCCTATGCGTGAA R: GCGGCTTGAATGTTTGTC	109
*HO-1*	F: ACAGATGGCGTCACTTCG R: TGAGGACCCACTGGAGGA	128
*SREBP1c*	F: GCCATGGATTGCACATTG R: TGTGTCTCCTGTCTCACCCC	102
*SCD1*	F: CCTTAACCCTGAGATCCCGTAGA R: AGCCCATAAAAGATTTCTGCAA	237
*FAS*	F: GGACATGGTCACAGACGATGAC R: GGAGGCGTCGAACTTGGA	194

## Data Availability

The data presented in this study are openly available in [SRA] at [https://www.ncbi.nlm.nih.gov/sra], reference number [PRJNA1198395].
